# Top-down modulation of dichotic listening affects interhemispheric connectivity: an electroencephalography study

**DOI:** 10.3389/fnins.2024.1424746

**Published:** 2024-09-12

**Authors:** Osama Elyamany, Jona Iffland, Denise Lockhofen, Saskia Steinmann, Gregor Leicht, Christoph Mulert

**Affiliations:** ^1^Centre of Psychiatry, Justus Liebig University Giessen, Hessen, Germany; ^2^Centre for Mind, Brain and Behaviour, Marburg, Hessen, Germany; ^3^Department of Psychiatry and Psychotherapy, University Medical Centre Hamburg-Eppendorf, Hamburg, Germany

**Keywords:** dichotic listening, interhemispheric connectivity, top-down modulation, auditory hallucinations, EEG, lagged-phase synchronisation

## Abstract

**Introduction:**

Dichotic listening (DL) has been extensively used as a task to investigate auditory processing and hemispheric lateralisation in humans. According to the “callosal relay model,” the typical finding of a right ear advantage (REA) occurs because the information coming from the right ear has direct access to the left dominant hemisphere while the information coming from the left ear has to cross via the corpus callosum. The underlying neuroanatomical correlates and neurophysiological mechanisms have been described using diffusion tensor imaging (DTI) and lagged phase synchronization (LPS) of the interhemispheric auditory pathway. During the non-forced condition of DL, functional connectivity (LPS) of interhemispheric gamma-band coupling has been described as a relevant mechanism related to auditory perception in DL. In this study, we aimed to extend the previous results by exploring the effects of top-down modulation of DL (forced-attention condition) on interhemispheric gamma-band LPS.

**Methods:**

Right-handed healthy participants (*n* = 31; 17 females) performed three blocks of DL with different attention instructions (no-attention, left-ear attention, right-ear attention) during simultaneous EEG recording with 64 channels. Source analysis was done with exact low-resolution brain electromagnetic tomography (eLORETA) and functional connectivity between bilateral auditory areas was assessed as LPS in the gamma-band frequency range.

**Results:**

Twenty-four participants (77%) exhibited a right-ear advantage in the no-attention block. The left- and right-attention conditions significantly decreased and increased right-ear reports, respectively. Similar to the previous studies, functional connectivity analysis (gamma-band LPS) showed significantly increased connectivity between left and right Brodmann areas (BAs) 41 and 42 during left ear reports in contrast with right ear reports. Our new findings notably indicated that the right-attention condition exhibited significantly higher connectivity between BAs 42 compared with the no-attention condition. This enhancement of connectivity was more pronounced during the perception of right ear reports.

**Discussion:**

Our results are in line with previous reports describing gamma-band synchronization as a relevant neurophysiological mechanism involved in the interhemispheric connectivity according to the callosal relay model. Moreover, we newly added some evidence of attentional effects on this interhemispheric connectivity, consistent with the attention-executive model. Our results suggest that reciprocal inhibition could be involved in hemispheric lateralization processes.

## 1 Introduction

**FIGURE 1:**
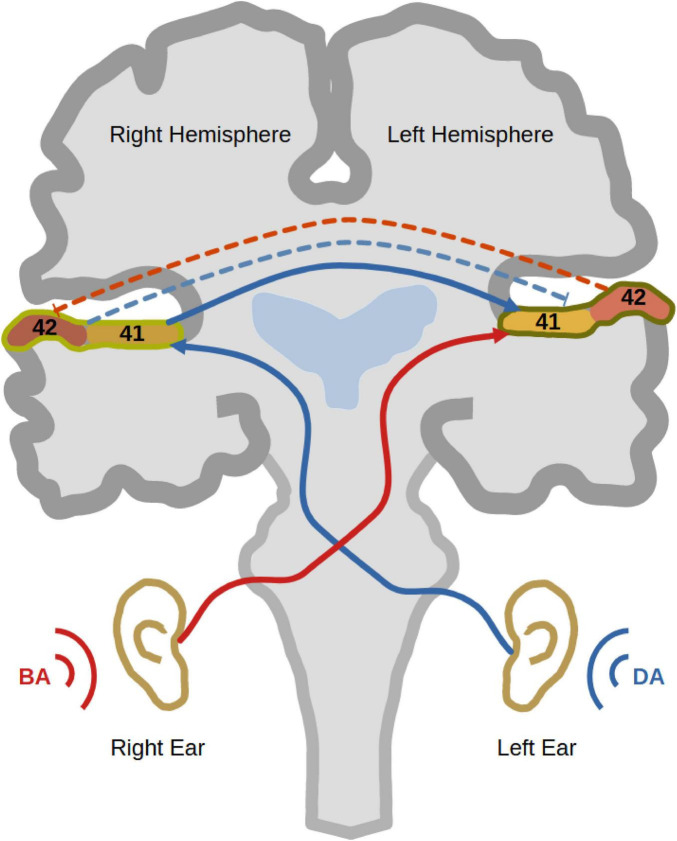
Dichotic listening. The figure shows a trial of dichotic listening where the syllable “BA” is presented to the right ear and the syllable “DA” is presented to the left ear. As well, it shows the trajectory of auditory information transfer to the left auditory cortex, where the auditory information from the right ear has direct access to the left dominant hemisphere (red). On the other hand, the auditory information presented to the left ear travels first to the right auditory cortex and then through the corpus callosum to the left hemisphere (blue). That explains why most right-handed participants show a right ear advantage (REA). During a dichotic listening experience, reciprocal inhibition between auditory cortices occurs to inhibit the processing of the ipsilateral sound at the level of auditory cortex (bidirectional dashed lines).

Dichotic listening (DL) has been extensively used as a task to investigate auditory processing and hemispheric lateralisation in humans for more than six decades (Broadbent, 1956). It involves the simultaneous presentation of two different sounds to both ears via headphones. While one sound is presented to the right ear, a different sound is presented simultaneously to the left ear. Then, the participant is asked to report which sound they perceived (Figure 1). People with left-hemispheric dominance normally tend to report more sounds from the right ear than sounds from the left (Kinsbourne, 1970; Cheatham, 1990). This observation is typically called the “right ear advantage” (REA), first discovered in Kimura (1961a,1961b). According to the “callosal relay model,” the REA occurs because the information coming from the right ear has direct access to the left dominant hemisphere (Zaidel, 1983). On the other hand, for the left ear sound to be perceived, auditory information from the left ear reaches first the right hemisphere, from which it travels through the corpus callosum to the dominant left hemisphere. In other words, the left ear report necessitates enhanced interhemispheric connectivity between both auditory cortices (Zaidel, 1983; Hugdahl and Westerhausen, 2016). Interestingly, this enhanced interhemispheric communication was structurally [using diffusion tensor imaging (DTI)] and functionally [using electroencephalography (EEG)] verified (Westerhausen et al., 2006; Westerhausen et al., 2009a; Steinmann et al., 2018a). Another phenomenon, which contributes to REA, is the reciprocal inhibition between both auditory cortices during DL (Brancucci et al., 2004). Since right-handed persons commonly, but not always, possess left-hemispheric dominance, most of them show the typical REA (Cheatham and Herbig, 1992).

Like most psychological tasks involving attention, DL can be modulated by either top-down or bottom-up mechanisms (Andersson et al., 2008). While top-down modulation involves attentional instructions, bottom-up modulation comprises manipulations of sound properties such as sound intensity (Westerhausen et al., 2009b). Attention instructions during top-down modulation are conducted by asking participants to attend actively to either the left or the right ear during DL, resulting in reduction or enhancement of REA, respectively (Bryden et al., 1983). Such instructions allocate higher cognitive resources to interact with the bias toward the right ear sound (i.e., REA) either to increase or decrease it. This interaction is called the “attention-executive model” (Hugdahl and Westerhausen, 2016). Deliberately attending to the right ear increases the REA, while left ear attention decreases it.

Top-down modulation of DL has been investigated using different imaging techniques: functional magnetic resonance imaging (fMRI), EEG, magnetoencephalography (MEG), and functional near-infrared spectroscopy (fNIRS), to describe the neurobiological correlates of its behavioural outcomes. A recent fMRI study observed more activation in the auditory temporal cortex on the left side compared with the right side. However, no certain activation patterns were found while comparing the forced left/right and non-forced conditions to each other (Kazimierczak et al., 2021). Previous fMRI studies suggested activation of the auditory cortex contralateral to the attended ear (O’Leary et al., 1996; Jäncke et al., 2001), while other studies highlighted the involvement of frontal regions in the attentional control (Pugh et al., 1996; Thomsen et al., 2004; Kompus et al., 2012). MEG Studies showed similar results, as an MEG-based study showed higher ipsilateral synchronization in the frontal region (Gootjes et al., 2006). Additionally, the contralateral auditory cortex was more activated during the forced attention conditions (Alho et al., 2012), while the response of the auditory cortex was higher during the active attention compared to passive attention (Tanaka et al., 2021). A single fNIRS study added to the evidence of frontal activation, which was exaggerated upon higher cognitive demand in the left attention condition in comparison to the non-forced condition (Eskicioglu et al., 2019). Finally, the attentional input to lower regions within the auditory system was found to be more implicated in the attentional control of DL, according to a modelling study, demanding more research on the role of auditory temporal cortices (Kuo et al., 2022). These results not only prove the role of the frontal regions in attentional control, but they suggest also the implication of the temporal auditory cortices in this process. Applying EEG, many previous studies discovered other processes involved in attentional control during DL (Payne et al., 2017; Dahl et al., 2019; Teoh and Lalor, 2019; Dahl et al., 2020). An interesting finding was the detection of right frontotemporal alpha modulation only during the right attention condition, compared to passive listening (Payne et al., 2017). Given the callosal relay model, it might be needed to analyse interhemispheric interaction between both auditory cortices via EEG, especially after giving attentional instructions (Berretz et al., 2020). However, only one study investigated interhemispheric interaction during top-down modulation with attention instructions (Razumnikova and Vol’f, 2007), but the study focused on the interaction between frontal and/or parietooccipital areas. To the best of our knowledge, no previous studies focused on the interhemispheric communication between both auditory cortices during top-down modulation of DL.

Interestingly, during the non-forced condition, EEG helped to reveal interesting results based on the above-mentioned interhemispheric connectivity between both auditory cortices (Razumnikova and Vol’f, 2007; Steinmann et al., 2014a; Solcà et al., 2016; Steinmann et al., 2017; Steinmann et al., 2018b; Steinmann et al., 2018a; Thiebes et al., 2018; Momtaz et al., 2021). The findings showed that the left ear reports were associated with increased interhemispheric communication in the gamma range (Steinmann et al., 2014a), consistent with the callosal relay model (Hugdahl and Westerhausen, 2016). Additionally, effective connectivity analysis revealed increased right-to-left interhemispheric connectivity only during left precepts (Steinmann et al., 2018b). These results were further substantiated by studies using diffusion tensor imaging showing anatomically stronger interhemispheric connections (Westerhausen et al., 2006; Westerhausen et al., 2009a; Steinmann et al., 2018a). Therefore, examining the interhemispheric communication during top-down modulation of DL would help detect the auditory information transfer during the different attention conditions. In this regard, the previous results did not highlight any relevant difference between the primary and the secondary auditory cortices, while the later could be more involved in integrating the attentional control of auditory processing (Grady et al., 1997; Johnsrude et al., 2002).

No study, so far, addressed attentional control on the neurophysiological mechanisms underlying the callosal relay model in the DL. Accordingly, we aimed to apply a top-down modulation of DL and examine its corresponding effects on interhemispheric connectivity between left and right auditory cortices. We hypothesised that REA would result from non-modulated trials of DL. Upon giving instructions to attend to the right or left ear, we expected to see an increase or decrease in REA, respectively. Using EEG and computing functional connectivity measures between auditory cortices, we extended our hypothesis to predict increased interhemispheric connectivity during left ear reports. More interestingly, we focused on the effects of the attentional shift, either to the left or to the right ear, on this interhemispheric connectivity. Additionally, we explored the interhemispheric connectivity at the primary and the secondary auditory levels separately, to investigate their individual roles during attentional control.

## 2 Materials and methods

We recruited healthy participants to perform a DL task with top-down modulation. During this task, EEG was simultaneously recorded to evaluate the functional connectivity between both the left and right auditory cortices of the brain during offline analysis.

### 2.1 Participants

This work included the recruitment of human participants after signing an informed consent. The whole work was carried out according to the approval of the ethical committee at the Faculty of Medicine (FB 11 Medizin), Justus-Liebig University Giessen, registered with the project number “AZ 34/19” in May 2019. Native German speakers were invited to take part in the study at the research lab of the Department of Psychiatry at Justus Liebig University Giessen in Germany. The inclusion criteria included both genders, any age between 18 and 65 years and right-handedness. Exclusion criteria were left-handedness, hearing disability, any neurological or psychiatric disorder or taking medications that could affect the brain. According to these criteria, 33 participants (17 females) took part in the study. Two male participants were excluded later: one because of a neurological disorder and the other due to intake of thyroxine. The remaining 31 participants (17 females) were considered for analysis.

### 2.2 Questionnaires

After having given informed consent, each participant filled in three questionnaires: the Edinburgh Handedness Inventory (EHI) (Oldfield, 1971), a questionnaire for sociodemographic data and the schizotypal personality questionnaire (SPQ) (Raine, 1991).

### 2.3 Audiometry

The hearing ability of the participants was investigated using Home Audiometer Hearing Test (Esser Audio Software, 2023). Three different frequencies of sound (500, 1000, and 2000 Hz) were tested. Participants were excluded when they could not hear sounds below 15 dB or when the hearing difference between both ears was more than 9 dB.

### 2.4 Dichotic listening task

Participants performed a special task of DL in a soundproof and electrically shielded room. They listened to sounds through Sennheiser HDA 300 headphones (Sennheiser Audiometers Headphones, 2021). We used one of the most typical DL paradigms, which is the consonant-vowel (CV) version (Shankweiler and Studdert-Kennedy, 1967). Not only is it a simple paradigm, but also its stimuli are devoid of any emotional or semantic valence. The typical CV paradigm contains syllables made of a consonant sound combined with a vowel sound. For example, the Bergen CV paradigm consists of six different consonants (b - d - g - p - t - k) combined with the vowel /a/ to form six syllables (ba - da - ga - pa - ta - ka) (Hugdahl and Andersson, 1986). In this study, we used a German version of the Bergen CV paradigm, where a male German native speaker pronounced the syllables, while we kept the loudness at 70 dB. Three of these syllables are voiced (ba – da – ga) while the other three (pa - ta - ka) are their corresponding unvoiced syllables. Combining these six syllables into sound pairs gives rise to 36 pairs to be presented through headphones (i.e., one syllable for each ear). Considering a dichotic listening paradigm, six identical pairs are excluded resulting in 30 dichotic pairs. However, due to the difference in pronunciation between voiced and unvoiced syllables, it is advisable to use only homologous pairs: voiced-voiced and unvoiced-unvoiced ones (Westerhausen and Kompus, 2018). Therefore, the paradigm can be reduced to twelve pairs of either voiced (ba-ga, ba-da, da-ba, da-ga, ga-da and ga-ba) or unvoiced nature (pa-ka, pa-ta, ta-pa, ta-ka, ka-ta and ka-pa). During each trial, one pair of these 12 combinations was presented simultaneously to both ears: one syllable to the right ear and the other syllable to the left ear, respectively. Before the experiment, participants were not informed that they would hear two different sounds. They were instructed to listen and report after each trial, which sound they heard from the six different syllables.

Each participant had to finish three different blocks with each containing 121 trials. The trials were randomised within each block using Python 3 (Python.org, 2021) so that no pair would be repeated within two successive trials. Each of the selected twelve pairs was repeated ten times within each block. A test (ba-ba) pair was added in the middle of each block. A pause of 1 min was inserted between blocks. During the first block, participants were instructed to listen and report the best perceived sound. However, during the second and the third, they were asked to attend to either the left or the right ear, respectively. Therefore, each participant was subject to three different conditions: no-attention (NA), left-attention (LA), and right-attention (RA). The order of instructions during the second and third blocks was randomised between subjects.

**FIGURE 2:**
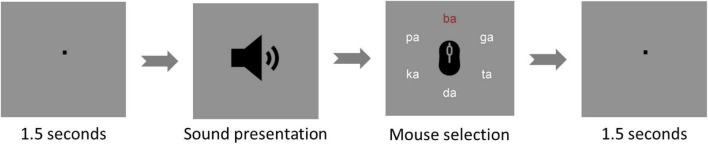
Dichotic listening trial. The figure shows one of the dichotic listening trials used during the experiment. The trials start with a fixation point for 1.5 s and then, the sound is presented. Afterward, the participant must select - with a left click of the mouse - the best-heard syllable out of six different options (ba - da - ga - pa - ta - ka). After confirmation of the selection with a right click, the next interval of 1.5 s is presented.

The task was run on a monitor at a 1-m distance from the eyes of participants using Presentation^®^ software from Neurobehavioral Systems (Version 20.1, Neurobehavioral Systems, Inc., Berkeley, CA, USA).^1^ At the beginning of each trial, a fixation sign was presented in the middle of the screen for 1 s followed by the auditory presentation of one syllable pair. Afterward, the six used syllables were shown in a circular structure, from which participants could choose the perceived sound. Participants could navigate through the circle and then confirm their choice by clicking the left and the right mouse buttons, respectively (Figure 2). Therefore, after each trial, participants could choose one of two correct reports (i.e., right ear or left ear syllables) or mistakenly select an incorrect report. If a participant showed incorrect reports for more than 25% of the trials, their dataset would be excluded from the analysis.

The log files exported from the Presentation software were analysed using Python 3 (Python.org, 2021). After excluding the incorrect reports, the numbers of right and left ear reports (RE and LE) per block were calculated. From these values, a laterality index (LI), which is a measure of REA, was computed for each block using the following formula:


LI=(RE-LE)/(RE+LE)×100.

### 2.5 EEG recording

The electrical activity of the brain was measured using a 64-channel EEG device. The device contained two (BrainAmp DC) amplifiers: each gave rise to 32 active channels (Brain Products, Munich, Germany). The electrodes were placed over the scalp according to an extended 10/20 configuration. The head circumference of each participant was measured and then a cap with a suitable size and the same configuration was applied to their head (ActiCaps, Brain Products, Munich, Germany). An electro-conductive gel was spread between the electrodes and the scalp to ensure an impedance level below 5 KΩ (ActiCaps, Brain Products, Munich, Germany). Reference and ground electrodes were put on the FCz and AFz positions of the cap, respectively. Four electrodes were dedicated to electrooculography (EOG): two superior and inferior vertical electrodes (VEOGS and VEOGI) and two right and left horizontal electrodes (HEOGR and HEOGL). Brain Vision Recorder software version 1.21.0303 was used for recording with a sampling rate of 1000 Hz (Brain Products, Munich, Germany). Before running the DL task, a 5-min resting EEG was recorded. The task was then run with simultaneous EEG recording.

### 2.6 EEG pre-processing

Brain Vision Analyzer software version 2.2 was used to pre-process the EEG datasets (Brain Products, Munich, Germany). At first, a radial EOG (REOG) channel was created to detect micro-saccades using the following formula (Keren et al., 2010):


REOG=

{(VEOGS+VEOGI+HEOGR+HEOGL)/4)}-Pz.

All EEG channels were then referenced to a new common average reference formed from all EEG channels without the EOG electrodes while retaining FCz as a normal channel. Afterward, an infinite impulse response (IIR) filter was applied to cut off all frequencies below 30 Hz and more than 120 Hz with an order of two. The filtered data were later down-sampled to 500 Hz. To remove the visually obvious artefacts, the whole dataset was inspected semi-automatically. To get rid of special sources of contamination (eye, muscle and sweat artefacts), an independent component analysis was run. The resulting components were scanned according to their topography, frequency composition and the patterns of coarse eye movements and micro-saccades. After removing the suspicious components, the dataset of each participant was segmented around the time point of sound presentation. Each segment extended from 200 ms before to 824 ms after the stimulus presentation. Then, a baseline correction was applied for each segment. After excluding the segments of incorrect reports, the remaining segments were exported for connectivity analysis.

### 2.7 functional connectivity analysis

Depending on EEG data, functional connectivity between distant brain regions stands as a valid method to measure the interaction between these regions (Fingelkurts et al., 2005). One measure of functional connectivity between brain regions is lagged-phase synchronisation (LPS) (Wilmer et al., 2012; Olcay and Karaçalı, 2019). Unlike other measures of functional connectivity, LPS cancels out the effects of artificial artefacts and low spatial resolution (Stam et al., 2007). As well, it could offer clues concerning the spectral domain of brain connectivity, for example, using exact low-resolution brain electromagnetic tomography (eLORETA) (Pascual-Marqui, 2007; Pascual-Marqui et al., 2011).

Afterward, the exported segments were organised into six groups of segments [each attention condition (NA, LA and RA) had two types of reports (LE and RE)]. At the individual level, the numbers of segments per group were matched to obtain comparable connectivity values. So, the six groups of segments from the same participant included the same number, which was equal to the number of segments within the group with the smallest number of segments. To avoid any bias from the sample size, any subject with a number of segments below 20 was excluded from the connectivity analysis.

**FIGURE 3:**
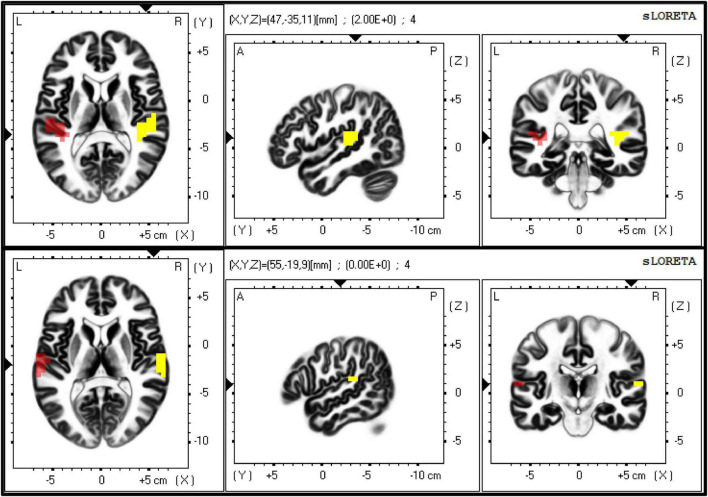
The figure shows the exact localisation of Brodmann areas (BAs) 41 **(upper panel)** and 42 **(lower panel)**, as extracted by eLORETA. These two regions of interest (ROIs) were used as seeds to calculate the lagged-phase synchronisation (LPS) between primary and secondary auditory cortices on both sides, respectively.

LORETA software was used (eLORETA, 2021). First of all, a transformation matrix was created according to the configuration of recording electrodes using the exact LORETA method. To assess connectivity between both right and left auditory cortices, Brodmann areas (BAs) 41 (i.e., primary auditory cortex) and 42 (i.e., secondary auditory cortex) on both hemispheres were selected as regions of interest (ROIs) within the computed transformation matrix (Figure 3).

For our eLORETA connectivity analysis, LPS was used, where the frequency band of interest was set to the gamma band (30 Hz – 100 Hz), according to previous studies (Steinmann et al., 2014a). All exported segments belonging to one of the six groups gave rise to one average LPS value, calculated from a specific time interval. In order to have reasonable time resolution, we divided the whole time window of segments (−200 to 824 ms) into ten equal intervals to calculate LPS values, each consisting of 100 ms. So, for each participant, we ended up with one LPS value for each condition/report (e.g., LA-LE for left ear reports during left attention) for each 100 ms interval.

### 2.8 Statistical analysis

For descriptive purposes, mean and standard deviation (SD) were used with continuous data. For categorical data, numbers and percentages were calculated. All figures of comparisons show mean and 95% confidence intervals.

For inferential statistics, one-way repeated-measures ANOVA tested the difference in laterality index between different conditions (NA, LA and RA). To test LPS differences between both LE and RE, a paired-sample *t*-test was used. In the case of condition comparison (NA, LA and RA), one-way ANOVA was suitable. To compare LPS values between different conditions (NA, LA and RA) and reports (LE and RE), a two-way repeated-measures ANOVA (Condition x Report) was applied for each 100ms-interval. The Greenhouse-Geisser correction was applied whenever Mauchly’s sphericity test showed significance. A Student’s paired-sample *t*-test was selected for two-variable comparisons with normal distributions, while the Wilcoxon signed-rank version was considered in the case of abnormal distributions. Normality was investigated via the Shapiro-Wilk test and Q-Q plots.

Both JASP (JASP Team, 2022) and SPSS (IBM Corp, 2022) software packages were used throughout the analysis. A significant result would mean a *p*-value below 0.05. The full statistical analysis is available in the “Supplementary material 1 inferential statistics”.

## 3 Results

### 3.1 Sociodemographic data

We included 31 participants (17 females) for further analysis. Their age ranged from 20 to 38 years old (mean = 24.161, SD = 4.124). All of them were right-handed according to EHI. No participant reached the threshold of clinical significance for schizotypal personality (i.e., a total score of 41) (Raine, 1991).

### 3.2 Audiometry

All participants, who took part in the study, were able to hear sounds below 15 dB and their interaural differences were less than 9 dB.

### 3.3 Dichotic listening task

All participants reported correct reports for more than 75% of all 363 trials (mean = 330.258, SD = 19.026), so the behavioural analysis included all of them. During the first condition NA, 24 (77 %) participants exhibited a positive LI (i.e., REA).

**FIGURE 4:**
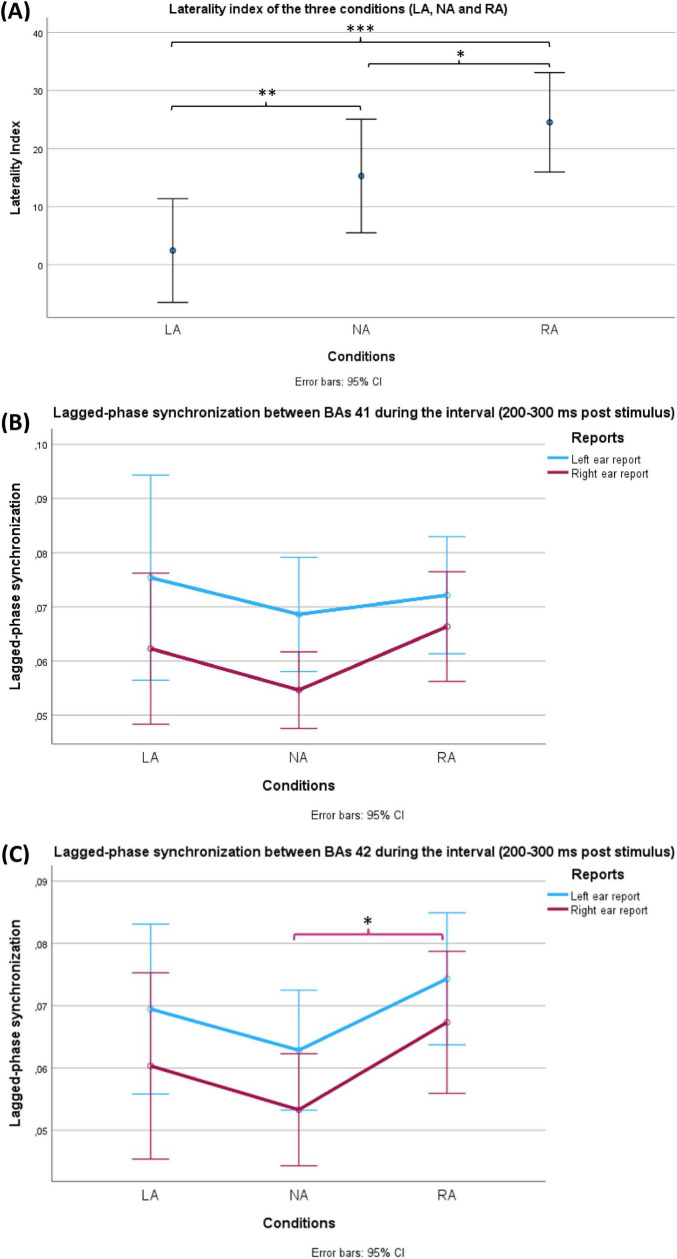
Behavioural **(A)** and EEG **(B,C)** results of the current study. **(A)** Shows a comparison between the laterality indices during the three conditions (LA: left-attention, NA: No-attention and RA: right-attention). The bars represent standard 95% confidence intervals. Using repeated measures ANOVA, the conditions factor showed significance (*p* < 0.001). All pairwise comparisons were significant after Holm correction. **(B,C)** Show a comparison of lagged-phase synchronisation (LPS) values between Brodmann areas (BAs) 41 and 42 of the groups of two factors, respectively. LPS values represent the interval (200 ms – 300 ms after the stimulus) between BAs 41 and 42 of the groups of two factors: conditions (LA, NA and RA) and reports (LE: left ear reports in blue and RE: right ear reports in red). The bars represent 95% confidence intervals. **(B)** LPS during LE was significantly higher than LPS during RE (*p* = 0.015) in the case of BAs 41. **(C)** LPS during LE was significantly higher than LPS during RE (*p* = 0.03) in the case of BAs 42. In the same case, the conditions factor (NA, LA and RA) reached a significance level (*p* = 0.048) where a *post hoc* test showed significantly higher LPS during RA in comparison with NA (*p* = 0.042). Only during RE, RA revealed significantly higher LPS compared with NA (*p* = 0.04). On the other hand, the corresponding comparison during LE did not reach the significance level (*p* = 0.051). The asterisk (*) is used to indicate the statistical significance (*p < 0.05, **p < 0.01, ***p < 0.001), while the error bars represent the confidence intervals (CI).

Upon applying one-way repeated-measures ANOVA on these laterality indices of the three conditions (NA, LA and RA), the analysis yielded a significant within-subjects effect (df = 1.580, *F* = 15.803, *p* < 0.001, η^2^p = 0.345). Post hoc test with Holm correction revealed that the three conditions differed significantly among participants in terms of laterality index (LA VS NA: *t* = −3.254, Cohen’s d = −0.517, *p* = 0.004; NA VS RA: *t* = −2.343, Cohen’s d = −0.373, *p* = 0.022; LA VS RA: *t* = −5.597, Cohen’s d = −0.890, *p* = 1.715 × 10−6) (Figure 4A).

### 3.4 EEG and connectivity results

One participant had a matching number of samples equal to eight, so his datasets were excluded from further connectivity analysis.

A) LPS between BAs 41:

Two-way repeated-measures ANOVA was conducted to compare the groups of two factors: conditions (NA, LA and RA) and reports (LE and RE) at each interval level (Figure 4B). The test showed a significant effect (df = 1, *F* = 6.732, *p* = 0.015, η^2^p = 0.188) only for reports (LE VS RE) and only at the interval 200 ms – 300 ms after stimulus presentation. This significance indicates that LE showed significantly higher LPS than RE in all three conditions.

B) LPS for BA 42:

At each interval, two-way repeated-measures ANOVA was conducted to compare the groups of two factors: conditions (NA, LA and RA) and reports (LE and RE). The test showed a significant effect for both reports (LE VS RE) (df = 1, *F* = 5.204, *p* = 0.03, η^2^p = 0.152) and conditions (NA, LA and RA) (df = 2, *F* = 3.211, *p* = 0.048, η^2^p = 0.100) at the interval 200 – 300 ms after the stimulus (Figure 4C). The former significance meant that LE showed significantly higher LPS than RE in all three conditions. A *post hoc* test with Holm correction was applied to compare the three conditions (NA, LA and RA). Only RA revealed a significant difference in comparison with NA (*t* = −2.532, Cohen’s d −0.406, *p* = 0.042) (Figure 4C). To explore which side of ear reports resulted in the significant difference in LPS between RA and NA, two paired-sample Student’s *t*-tests were applied (NA LE VS RA LE, and NA RE VS NA RE). In comparison to NA, RA showed significantly higher LPS values only during RE (*t* = −2.153, Cohen’s d = −0.393, *p* = 0.04). On the other hand, the corresponding comparison during LE did not reach the significance level (*t* = −2.032, Cohen’s d = −0.371, *p* = 0.051).

## 4 Discussion

### 4.1 Summary of results of the current study

In the current study, a top-down modulation was applied to a dichotic listening task using three attention instructions: no, left ear and right ear attention instructions (NA, LA and RA) with simultaneous EEG recording. LIs were computed from behavioural datasets of the three conditions while gamma-range LPS values were calculated from EEG data to compare the conditions (NA, LA and RA) and reports (LE and RE). Similar to previous results, 24 (77 %) participants exhibited a positive LI (i.e., REA) during the neutral NA condition. As well, all participants on average showed the typical REA (i.e., more reports from the right ear). LA and RA conditions managed to significantly decrease and increase REA, respectively.

In accordance with the previous literature, LPS between BAs 41 and between BAs 42 on both hemispheres revealed significantly more connectivity between the ROIs during LE than during RE at the interval of 200 ms to 300 ms after stimulus. Our novel results showed a discrepancy between BAs 41 and BAs 42 in terms of interhemispheric LPS during top-down modulation of DL. For the same interval, RA and LA showed increased connectivity between only BAs 42 in contrast to NA. However, only the difference between RA and NA was statistically significant. This increase during RA, in comparison to NA, could be detected only in the case of RE, but not LE.

### 4.2 Results of the right ear advantage (replication of previous studies)

The current study showed behavioural results during NA, which are in line with previous literature (Westerhausen et al., 2015a; Westerhausen et al., 2015b; Westerhausen and Kompus, 2018). Our study showed that 77 % of a sample of right-handed participants exhibited REA, quite close to the pool’s results (70.6 % - 75.3 %). To some extent, this right ear advantage can be explained by the callosal relay model (Zaidel, 1983). In that sense, left-hemispheric dominance, in most right-handed people, promotes bias to perceive the right ear sound easier than the left ear one (Westerhausen and Hugdahl, 2008). However, the callosal relay model cannot fully explain REA since the percentage of left-hemispheric dominance in the right-handed population is 96% (Rasmussen and Milner, 1977). This yields a difference of around 20% of right-handed individuals who do not show REA. To further explain this observation, another model (the attention-executive model) is introduced, where attentional control can interact with the built-in REA (Hugdahl and Westerhausen, 2016). This model predicts that the participants would report more from the ear they attend to. This process might interfere with the built-in right ear advantage, notably when the participants deliberately focus on the left ear. Although the participants were not instructed to attend to any ear during NA, they might idiosyncratically or randomly pay attention to one ear or another from trial to trial. This would lead to intra-individual, trial-to-trial fluctuations and therefore interfere with the right ear advantage. In addition to this interference, other stimulus-related factors (stimulus parameters such as the phonological properties) could contribute to this deviation from the right ear advantage (Westerhausen and Kompus, 2018).

### 4.3 Results of top-down modulation of dichotic listening (replication of previous studies)

As expected, this study showed REA during the NA condition, which was attenuated and potentiated during LA and RA conditions, respectively. This observation stands as a replicable finding upon top-down modulation of DL using attentional instructions (Hiscock and Kinsbourne, 2011). Again, the attention-executive model can justify the attentional effects on DL (Hugdahl and Westerhausen, 2016). Moreover, this proves that intra-individual inter-trial variation takes place within the same individual and it is not an absolute advantage of one ear per individual. In other words, the momentary interaction between higher cognitive control and the anatomical bias of REA dictates which ear sound would take advantage in each trial. According to the attention theory, when two competing stimuli reach the brain, one would be attenuated and the other would be perceived due to the limited capacity of the brain (Hiscock and Kinsbourne, 2011). Considering this theory in the DL context, one sound is ignored, while the other is further processed at higher levels. By default, a bias is dedicated to the right ear sound in the case of left-hemispheric dominance. However, attentional conditions, either deliberately or stochastically, can play a role in controlling the competition between two different sounds. In the case of uncontrolled attention (i.e., free-report condition, like NA condition), the attentional resources can fluctuate between the right and left ears. This describes the inter-trial variability during NA. On the other hand, when attention instructions are given, deliberate allocation of attentional resources to the left ear (LA condition) would compete with the lower level of hemispheric asymmetry and attenuate the built-in REA. In the case of right-attention instructions, the attentional processes would further potentiate the bias toward the right ear.

### 4.4 EEG results during the left and right ear reports (replication of previous studies)

Based on the callosal relay model, for the left ear sound to be perceived, the auditory information of the left ear should travel first to the right hemisphere, from which it should cross through the corpus callosum to the dominant left hemisphere (Steinmann et al., 2014b). Thus, it is a must to a establish connection between both auditory cortices to perceive the left sound, which was proven in subjects with the surgical section of commissures (Sparks and Geschwind, 1968). Therefore, it is expected that LE should show higher connectivity between both auditory cortices than RE. In accordance with this theory, our study managed to show more LPS during LE than during RE in all conditions, more specifically after 200 ms of stimulus presentation. This increase was observed between both BAs 41 and BAs 42 on both sides. These results go along with previous similar results of increased connectivity values during LE (Steinmann et al., 2014a; Steinmann et al., 2018b; Steinmann et al., 2018a). Additionally, this interhemispheric communication, in the form of gamma-band synchrony, was shown to be associated with structural components using diffusion tensor imaging (Steinmann et al., 2018a). Although every study showed different time points, at which the significant LPS difference is found, this can be explained by different technical equipment and delivery of stimuli. All of these studies addressed LPS within the gamma range, whose synchronisation is thought to be essential for the interaction between brain regions underlying cognitive functions and cortical computation (Fries, 2009). Noteworthily, these studies showed enhanced gamma-band interhemispheric connectivity at a more or less late time point after the stimulus presentation, which was the case in our results as well (200 ms – 300 ms after the stimulus). An interesting review has already addressed the timing pattern of the auditory processing at different stages based on event-related potentials (Joos et al., 2014). The review indicates that top-down modulation is mostly synchronised with P300 component. Since dichotic listening, in contrast to diotic listening, involves two competing sounds, it should exhibit a later stage of auditory processing integrating both top-down and bottom-up processes (Westerhausen et al., 2009b). In this regard, the frontal regions are involved in modulating the perception at the auditory cortices and their interhemispheric connectivity, notably the reciprocal inhibition. These mechanisms could explain the late timing of this enhanced interhemispheric connectivity.

### 4.5 Comparison of EEG results between brodmann areas (BAs) 41 and 42 (core results)

More surprisingly, RA (and in a non-significant way LA) showed higher LPS between both BAs 42, but not BAs 41 in comparison to NA. The discrepancy in findings between BAs 41 and BAs 42 could be attributed to their functional specificity. While BA 41 (i.e., primary auditory cortex) is responsible for primary properties of sounds such as frequency and intensity, BA 42 processes complex sounds and voices (Grady et al., 1997; Binder et al., 2000; Khalil, 2020). Therefore, BA 41 should show more activation for the fundamental properties of sounds, which could be translated into interhemispheric connectivity to perceive the left sound and block the right one. On the other hand, BA 42, as an auditory association hub, could be more affected by attentional input, and thus show a contrast between different attentional conditions (Grady et al., 1997). Another possibility could be the limited spatial resolution of EEG and eLORETA (Burle et al., 2015). An interesting new result was the significant difference between different attentional conditions, more specifically RA showed higher connectivity than NA. It is noteworthy that auditory cortices are affected by attentional instructions, especially BAs 42 (Grady et al., 1997; Johnsrude et al., 2002). As well, top-down modulation of DL affects frontal activity underlying attentional processes, which may, in turn, modulate auditory cortical activity (Gootjes et al., 2006). Based on that, during RA, attentional input to the auditory cortex is enhanced to promote the right ear sound, which is favoured by default. In such a case, this increased interhemispheric communication during RA might reflect higher right-to-left-hemispheric information transfer, which helps, in turn, to overcome the attentional input - concentrating on the right ear - especially during LE. Another explanation might be the reciprocal inhibition between bilateral auditory areas, where the left hemisphere extensively inhibits the right hemisphere information, notably during RE (Brancucci et al., 2004). Our results would support the second explanation, since the enhanced interhemispheric communication during RA, in comparison to NA, could be attributed to the contribution of RE, rather than LE. This agrees with the previous finding of enhanced parietal and right frontotemporal alpha modulation during RA (Payne et al., 2017). Moreover, during deliberate attention, sound processing was promoted within the hemisphere contralateral to the side of attention (O’Leary et al., 1996; Jäncke et al., 2001; Alho et al., 2012).

### 4.6 Auditory hallucinations outlook

The current findings may not only add to the current understanding of speech lateralisation but might also help to clinically investigate patients with schizophrenia, especially those with auditory hallucinations. Auditory hallucinations have been associated with increased interhemispheric communication during DL in either patients with schizophrenia, schizophrenia-like models, or individuals at clinical high-risk for psychosis (Steinmann et al., 2017; Thiebes et al., 2018; Langhein et al., 2023). These findings are consistent with the interhemispheric miscommunication theory of auditory hallucinations (Ćurčić-Blake et al., 2017; Steinmann et al., 2019; Weber et al., 2021). This disrupted circuitry can serve in the future as a potential neurostimulation target for non-invasive brain stimulation in patients with schizophrenia (Elyamany et al., 2021), as it has already been investigated in healthy participants with some promising results (Meier et al., 2019; Preisig et al., 2020; Preisig et al., 2021).

### 4.7 Limitations of the current study

Despite the new findings of this study, it possesses some limitations that could be improved in future research. First, we recruited a relatively small number of participants, so it would be recommended to replicate the experiment with a bigger sample size. Second, a limitation of this study is the limited spatial accuracy of the LORETA approach, although several cross-validation studies using simultaneous EEG and fMRI have suggested sufficient validity of the LORETA approach in general (Mulert et al., 2004). Third, LPS, though being valid for functional connectivity in general, does not give a clue about the direction of connectivity. Effective connectivity analysis should be applied to assess the directionality of information flow. Fourth, the results represent associative rather than causal relationships. However, some studies have already discovered the causal effect of interhemispheric connectivity and validated the callosal relay model (Zaidel, 1983).

## 5 Conclusion

During the neutral NA condition of DL, the right-handed population shows the typical right ear advantage. This is attributed to REA explained by the callosal relay model. The model shows that left ear information should cross to the dominant left hemisphere to be perceived, unlike right ear information, which has direct access to the dominant hemisphere. This REA could be manipulated by top-down modulation giving attentional instructions, which affect the behavioural outcome by increasing the reports from the ear attended to. RA and LA conditions managed to increase or decrease REA, respectively. Such manipulation is considered in terms of the attention-executive model, which implies an interaction between the attentional input to auditory cortices and the built-in anatomical REA.

Using EEG and computing LPS, LE showed higher interhemispheric connectivity between BAs 41 on both sides than RE in all conditions. This reflects the transfer of auditory information from the right to the left hemisphere according to the callosal relay model. Attentional condition RA showed more interhemispheric connectivity between BAs 42, compared to NA. This might result from reciprocal inhibition between bilateral auditory cortices, especially during RE. Finally, this study might help in the future to modulate interhemispheric connectivity between both auditory cortices aiming to reset abnormal communication during auditory hallucinations.

## Data Availability

The raw data supporting the conclusions of this article will be made available by the authors, without undue reservation.

## Footnotes

1
https://www.neurobs.com/
