# Corrigendum: Top-down modulation of dichotic listening affects interhemispheric connectivity: an electroencephalography study

**DOI:** 10.3389/fnins.2025.1579854

**Published:** 2025-03-11

**Authors:** Osama Elyamany, Jona Iffland, Denise Lockhofen, Saskia Steinmann, Gregor Leicht, Christoph Mulert

**Affiliations:** ^1^Centre of Psychiatry, Justus Liebig University Giessen, Hessen, Germany; ^2^Centre for Mind, Brain and Behaviour, Marburg, Hessen, Germany; ^3^Department of Psychiatry and Psychotherapy, University Medical Centre Hamburg-Eppendorf, Hamburg, Germany

**Keywords:** dichotic listening, interhemispheric connectivity, top-down modulation, auditory hallucinations, EEG, lagged-phase synchronisation

In the published article, there was an error. In two instances within the discussion section, we mistakenly reversed “RE” and “LE.”

A correction has been made to **Discussion section**, *4.1 Summary of results of the current study*, Paragraph 2. This sentence previously stated:

“This increase during RA, in comparison to NA, could be detected only in the case of LE, but not RE.”

The corrected sentence appears below:

“This increase during RA, in comparison to NA, could be detected only in the case of RE, but not LE.”

A correction has been made to **Discussion section**, *4.5 Comparison of EEG results between brodmann areas (BAs) 41 and 42 (core results)*. This sentence previously stated:

“Our results would support the second explanation, since the enhanced interhemispheric communication during RA, in comparison to NA, could be attributed to the contribution of LE, rather than RE.”

The corrected sentence appears below:

“Our results would support the second explanation, since the enhanced interhemispheric communication during RA, in comparison to NA, could be attributed to the contribution of RE, rather than LE.”

The authors apologize for this error and state that this does not change the scientific conclusions of the article in any way. The original article has been updated.

