# Supplementary material for: Top-down modulation of dichotic listening affects interhemispheric connectivity: an electroencephalography study
Source: Front Neurosci. 2024 Sep 12;18:1424746. doi: 10.3389/fnins.2024.1424746 (PMC11424531; doi:10.3389/fnins.2024.1424746)
Supplement: Supplementary file 1 [file Data_Sheet_1.PDF]

Supplementary Material 1

A) Behavioural Data (Dichotic Listening Task)

One-way repeated-measures ANOVA for the three conditions (LA: left-attention, NA: No-attention and RA: right-attention)

Within Subjects Effects

Cases	Sphericity Correction	Sum of Squares	df	Mean Square	F	p	η^2_p
Conditions	None	7635.483 [a]	2.000 [a]	3817.741 [a]	15.803 [a]	3.068×10^{-6} [a]	0.345
	Greenhouse-Geisser	7635.483	**1.580**	4833.970	**15.803**	**2.375×10^{-5}**	0.345
Residuals	None	14495.106	60.000	241.585			
	Greenhouse-Geisser	14495.106	47.386	305.892			

Note. Type III Sum of Squares

[a] Mauchly's test of sphericity indicates that the assumption of sphericity is violated ($p < .05$).

Assumption Checks

Test of Sphericity

	Mauchly's W	Approx. X^2	df	p-value	Greenhouse-Geisser ε	Huynh-Feldt ε	Lower Bound ε
Conditions	0.734	8.975	2	**0.011**	0.790	0.826	0.500

Post Hoc Tests

Post Hoc Comparisons - Conditions

		Mean Difference	SE	t	Cohen's d	p_{holm}
LA	NA	-12.847	3.948	-3.254	-0.517	**0.004** **
	RA	-22.097	3.948	-5.597	-0.890	**1.715×10^{-6}** ***
NA	RA	-9.250	3.948	-2.343	-0.373	**0.022** *

* $p < .05$, ** $p < .01$, *** $p < .001$

Note. P-value adjusted for comparing a family of 3

B) EEG Analysis(Lagged-phase synchronisation)

1) Brodmann areas (BAs) 41

Two-way repeated-measures ANOVA to compare the groups of two factors: conditions (LA: left-attention, NA: No-attention and RA: right-attention) and reports (LE: left ear and RE: Right ear) at the interval 200 ms – 300 ms after stimulus presentation.

Within Subjects Effects

Cases	Sum of Squares	df	Mean Square	F	p	η^2_p
Conditions	0.002	2	0.001	1.098	0.340	0.036
Residuals	0.058	58	0.001			
Reports	0.005	1	0.005	6.732	**0.015**	0.188
Residuals	0.023	29	8.033×10^{-4}			
Conditions ✻ Reports	6.053×10^{-4}	2	3.027×10^{-4}	0.567	0.570	0.019
Residuals	0.031	58	5.341×10^{-4}			

Note. Type III Sum of Squares

2) Brodmann areas (BAs) 42

Two-way repeated-measures ANOVA to compare the groups of two factors: conditions (LA: left-attention, NA: No-attention and RA: right-attention) and reports (LE: left ear and RE: Right ear) at the interval 200 ms – 300 ms after stimulus presentation.

Within Subjects Effects

Cases	Sum of Squares	df	Mean Square	F	p	η^2_p
Conditions	0.005	2	0.002	3.211	**0.048**	0.100
Residuals	0.044	58	7.602×10^{-4}			
Reports	0.003	1	0.003	5.204	**0.030**	0.152
Residuals	0.018	29	6.341×10^{-4}			
Conditions ✻ Reports	5.698×10^{-5}	2	2.849×10^{-5}	0.046	0.955	0.002
Residuals	0.036	58	6.247×10^{-4}			

Note. Type III Sum of Squares

Post Hoc Comparisons - Conditions

		Mean Difference	SE	t	Cohen's d	p_{holm}
LA	NA	0.007	0.005	1.355	0.217	0.361
	RA	-0.006	0.005	-1.177	-0.189	0.361
NA	RA	-0.013	0.005	-2.532	-0.406	**0.042** *

* p < .05

Note. P-value adjusted for comparing a family of 3

Note. Results are averaged over the levels of: Reports

Two paired-sample Student's t-tests were applied (NA LE VS RA LE, and NA RE VS NA RE) to explore which side of ear reports resulted in the significant difference in LPS between RA and NA.

Measure 1		Measure 2	t	df	p	Cohen's d
NA LE	-	RA LE	-2.032	29	0.051	-0.371
NA RE	-	RA RE	-2.153	29	0.040	-0.393
